# Artificial intelligence forecasting mortality at an intensive care unit and comparison to a logistic regression system

**DOI:** 10.31744/einstein_journal/2021AO6283

**Published:** 2021-09-29

**Authors:** Beatriz Nistal-Nuño

**Affiliations:** 1 Department of Anesthesiology Complexo Hospitalario Universitario de Pontevedra PontevedraPO Spain Department of Anesthesiology, Complexo Hospitalario Universitario de Pontevedra, Pontevedra, PO, Spain.

**Keywords:** Artificial intelligence, Gradient boosted decision trees, Intensive care units, MIMIC-III database, Mortality, Discrimination, Logistic regression

## Abstract

**Objective:**

To explore an artificial intelligence approach based on gradient-boosted decision trees for prediction of all-cause mortality at an intensive care unit, comparing its performance to a recent logistic regression system in the literature, and a logistic regression model built on the same platform.

**Methods:**

A gradient-boosted decision trees model and a logistic regression model were trained and tested with the Medical Information Mart for Intensive Care database. The 1-hour resolution physiological measurements of adult patients, collected during 5 hours in the intensive care unit, consisted of eight routine clinical parameters. The study addressed how the models learn to categorize patients to predict intensive care unit mortality or survival within 12 hours. The performance was evaluated with accuracy statistics and the area under the Receiver Operating Characteristic curve.

**Results:**

The gradient-boosted trees yielded an area under the Receiver Operating Characteristic curve of 0.89, compared to 0.806 for the logistic regression. The accuracy was 0.814 for the gradient-boosted trees, compared to 0.782 for the logistic regression. The diagnostic odds ratio was 17.823 for the gradient-boosted trees, compared to 9.254 for the logistic regression. The Cohen’s kappa, F-measure, Matthews correlation coefficient, and markedness were higher for the gradient-boosted trees.

**Conclusion:**

The discriminatory power of the gradient-boosted trees was excellent. The gradient-boosted trees outperformed the logistic regression regarding intensive care unit mortality prediction. The high diagnostic odds ratio and markedness values for the gradient-boosted trees are important in the context of the studied unbalanced dataset.

## INTRODUCTION

Accurate timely prediction of mortality before rapid patient deterioration may be paramount, specially at an intensive care unit (ICU).^(1)^ Deterioration of physiological and biochemical variables often precedes the clinical deterioration of patients at the ICU.^(2)^ Prediction of mortality at the ICU allows early interventions to be taken to remediate impending medical conditions, which could otherwise lead to a critical event and death.^(1)^

To anticipate patient deterioration at the ICU, several severity of illness scores have been developed. The Acute Physiology and Chronic Health Evaluation (APACHE) II system provides predictions for patient mortality, based on data collected at the ICU,^(3)^ and was refined as APACHE III, in 1991.^(4)^ A new version was published in 2006, APACHE IV, which added new variables and applied a different statistical method.^(5)^The Simplified Acute Physiology Score (SAPS) II was created to assess severity of disease of patients aged 15 years or more, admitted to ICU.^(6)^ The posterior SAPS III is a supplement to the SAPS II system.^(7)^

The Sequential Organ Failure Assessment (SOFA) score is used to assess a patient’s condition during their ICU stay, and degree of organs’ function.^(8)^ This score is based on six different scores, one for each system: central nervous, cardiovascular, respiratory, renal, liver, and coagulation. In the Logistic Organ Dysfunction System (LODS), physiological variables also assess dysfunction in six organ systems.^(9)^ The Oxford Acute Severity of Illness Score was developed by Johnson et al.^(10)^ The Mortality Prediction Model (MPM)-II system calculates the likelihood of hospital mortality for ICU patients.^(11)^

The majority of these prediction systems are linear scoring systems based on a weighted linear combination of patient features.^(1)^ These prediction tools assume that patient features are unrelated to each other, and, consequently, they cannot capture the complex interrelated physiology of patients.^(1)^ Intensive care unit prediction models, such as the APACHE, MPM, LODS, SAPS II and III, are based on multivariable logistic regression.^(12)^ Improved statistical methods have been evolved in this regard, like the recent system of Calvert et al.,^(1)^ which evaluated the correlations among grouped clinical predictor variables with all-cause mortality, within 12 hours, at the ICU, in addition to the analysis of patient measurement time trends using logistic regression.

One of the reasons for the low predictive power of many of the established scoring systems mentioned above lies in non-normality and non-linearity of the variables involved in modeling, as well as in nonlinear relations among physiologic variables and log odds of outcome, when using logistic regression.

Artificial intelligence (AI) has proved to be useful in this context, and a promising method to assess ICU mortality.^(12-14)^ Johnson et al., developed an ICU mortality prediction method, using a novel Bayesian ensemble learning algorithm. The proposed prediction method performed favorably, and had the potential to be utilized successfully for individual patient predictions.^(15)^

Johnson et al., compared AI, in the form of gradient-boosted decision trees (GBDT), to several types of logistic regression and models from the literature for real-time prediction of ICU patient mortality. The GBDT showed the highest area under the Receiver Operating Characteristic (ROC) curve (AUROC).^(16)^ Darabi et al., applied GBDT and deep neural networks to estimate the mortality risk of ICU patients.^(17)^

Kim et al., assessed whether the performance of various AI techniques, such as an artificial neural network, support vector machine and decision trees (DTs), outperformed the conventional logistic regression for ICU mortality prediction. They found that the DT algorithm slightly outperformed the other techniques.^(18)^

One AI method that has been successfully applied in this context is GBDT. Therefore, this research builds on the previous work of Calvert et al.,^(1)^ but using GBDT to compare the results.

## OBJECTIVE

To explore an artificial intelligence approach using gradient-boosted decision trees for prediction of all-cause mortality, at the medical intensive care unit, using the data of the Medical Information Mart for Intensive Care III database. This study compares the gradient-boosted trees performance to a logistic regression model built on the same platform, and the AutoTriage system for 12-hour mortality prediction at the medical intensive care unit. To date, such comparison has not been studied yet.

## METHODS

### Patient population and data extraction

The Medical Information Mart for Intensive Care (MIMIC)-III critical care database version v1.4. was used, a large database comprising de-identified comprehensive clinical data on individual patients admitted to ICUs at a large tertiary care hospital, the Beth Israel Deaconess Medical Center, in Boston, United States.^(19)^ Medical Information Mart for Intensive Care III contains data about adult patients admitted to ICUs between 2001 and 2012.^(19,20)^

**Figure 1:**
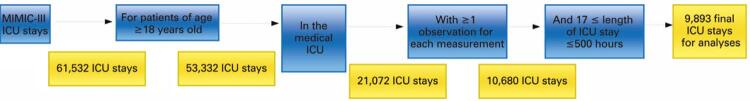
Patient data extraction steps from the Medical Information Mart for Intensive Care III critical care database

This study used a final dataset of 9,893 ICU-stay patient records from the MIMIC-III database, which were selected according to the data extraction steps outlined in figure 1. The patient exclusion process was performed as similar as possible to the one performed by Calvert et al.,^(1)^ to compare the results to the AutoTriage system. The selected subset consisted of the ICU-stay records of adult patients aged 18 years or more, admitted to the medical ICU, with at least one observation of each measurement for the specific parameters used in the analyses, and with a length-of-stay and survival from 17 hours to 500 hours after admission.

MIMIC III: Medical Information Mart for Intensive Care III; ICU: intensive care unit.

The number of ICU-stay records extracted at each step is the same as in the study,^(1)^ except for the last two steps. This is due to the fact that in the current work only temperature measurements in Celsius were collected, and this study uses a later version of the MIMIC-III database. Therefore, it was not possible to extract the exact same number of ICU-stay records for step 4, which also affected the step 5. However, the difference in the final number of ICU-stay records for analyses was very small, only 210 records out of the final number of 9,893 ICU-stay records collected for this study. The developed code in PostgreSQL language for the selection of the ICU stays is available at https://doi.org/10.7910/DVN/UMJVWA.^(21)^

Out of the final 9,893 ICU-stay records selected for analyses, 1,534 resulted in death during the ICU stay, and 8,359 resulted in ICU discharge with survival. That amounts to a prevalence of 15.5059% of ICU mortality.

### Factors associated with mortality

The 1-hour time-resolution physiological measurements collected during 5 consecutive hours from the 9,893 ICU-stay records comprised heart rate, pH, pulse pressure, respiratory rate, blood oxygen saturation, systolic blood pressure, temperature, and white blood cell count. These eight variables were chosen based on the study of Calvert et al.,^(1)^ to make the current results comparable, and because they are routine clinical parameters frequently measured at the ICU.

Data pre-processing was performed based on domain knowledge to remove erroneous recordings, like physiologically invalid values and unit errors. For a single missing hourly value, a replacement was calculated as the available value immediately preceding during the 5-hour window. For a missing value in the first hourly measurement, a replacement was calculated as the available value immediately following during the 5-hour window. For ICU stays where no data was collected for a particular parameter during the 5-hour window, the values applied were those in the normal range for that parameter.

This data was imported into the Konstanz Information Miner (KNIME) version 4.2.0 (KNIME AG, Zurich, Switzerland),^(22)^ in which the GBDT and logistic regression models were implemented, to execute the simulations. The study addressed how these models learned to represent and categorize these patients, based on their selected attributes into the categories of ICU-death or ICU-discharge, at a time 12 hours prior to the patient’s death or discharge.

**Figure 2:**
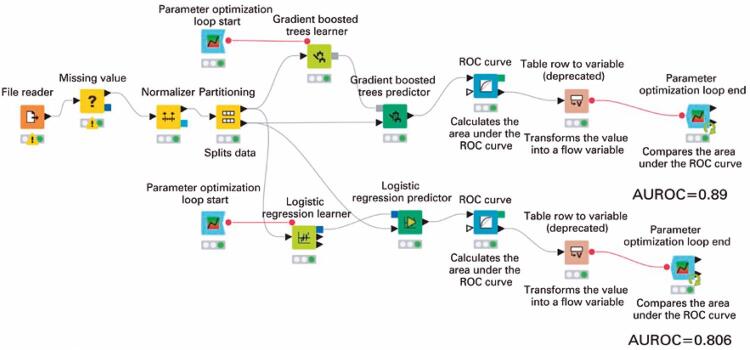
Konstanz information miner workflow. Print screen of the Konstanz information miner workflow used to build the gradient-boosted decision trees and logistic regression models

ROC: receiver operating characteristic; AUROC: area under the ROC curve.

The input dataset was randomly split into two partitions, 80% for train data and 20% for test data. This occurred after the normalizer node of KNIME normalized the values of all numerical input variables by Z-score normalization (Gaussian) (Figure 2).

### Artificial intelligence learning simulations

The used AI method of ensemble learning entails a combination of multiple AI models from supervised learning algorithms to obtain a more accurate overall model. The used ensemble technique is Boosting.^(22)^ Gradient-boosted decision trees is an ensemble model combining multiple sequential simple DTs into a stronger model, using a special form of boosting. At each iteration, a simple DT is fitted to predict the residuals of the current model, following the gradient of the loss function, and is added to the ensemble to improve the results from the previous model state, leading to higher performance after each iteration.^(22)^ The implementation follows the algorithm described in Friedman.^(23)^

It was implemented in KNIME by the GBDT learner node and the GBDT predictor node (Figure 2). The GBDT has the parameters mentioned in table 1, which were optimized through parameter optimization.^(22)^

**Table 1:** The best parameters found during the parameter optimization loops for the gradient-boosted decision trees model and logistic regression model

Parameters	Gradient-boosted decision trees	Logistic regression
Tree depth	7	
Number of models (DTs) to learn	1,175	
Learning rate	0.1	
Fraction of ICU records for each individual DT	0.5	
Attribute sampling (linear fraction) of patient features per tree node	0.1	
Solver		Iteratively reweighted least squares
Maximal number of epochs		2,140
Epsilon		0.01
Maximum number of iterations^*^	271	791
Number of rounds for early stopping^*^	108	188

^*^ The last two parameters are for the parameter optimization algorithm and not for the design of the models.

DT: decision tree; ICU: intensive care unit.

### Logistic regression learning simulations

Logistic regression is a statistical algorithm that models the relation between the input features and the categorical output classes by maximizing a likelihood function.^(22)^ It was constructed for the binary problem in this study in the same platform, to compare to the AI model developed, in addition to the comparison made to the AutoTriage system.^(1)^

It was implemented in KNIME by the logistic regression learner node and the logistic regression predictor node (Figure 2). The logistic regression has the parameters mentioned in table 1, which were optimized through parameter optimization.^(22)^

The technique of parameter optimization with a parameter optimization loop was employed to find the optimal parameters for the GBDT and logistic regression models. This was implemented in KNIME with the parameter optimization loop start node and the parameter optimization loop end node (Figure 2).

The parameters mentioned in table 1, controlled via flow variables, were chosen by an algorithm to maximize the AUROC for the outcome of ICU mortality prediction.^(22)^ The best values of the parameters found during the loops after several optimization simulations are shown in table 1. The remaining parameters were set to their default values.

### Performance measures

The metrics to evaluate the GBDT and logistic regression models and compare them to the AutoTriage were several accuracy statistics and the ROC curve with the AUROC. These measures were obtained after prediction of the 12-hour outcome class of the test set after training the models with the training set. The accuracy statistics evaluated were positive predictive value (PPV), negative predictive value (NPV), sensitivity, specificity, diagnostic odds ratio (OR), overall accuracy, Cohen’s kappa (CK), F-measure, Matthews correlation coefficient (MCC), and markedness (MK).

## RESULTS

**Figure 3:**
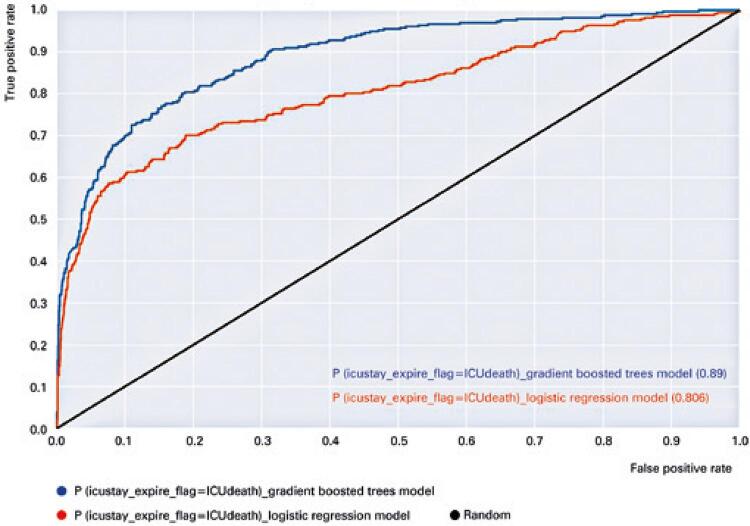
Receiver operating characteristic curve for 12-hour mortality prediction in the medical intensive care unit, for the gradient boosted decision trees and logistic regression models developed. The value representing whether the patient died in the intensive care unit or was discharged after the 12-hour interval was represented by the two-class target variable icustay_expire_flag

Figure 3 shows the ROC curves of the GBDT and logistic regression classifiers, which correspond to the AUROC values of 0.89 and 0.806, respectively (Table 2). The ROC curve is a graphical representation that displays the performance of a binary classifier as its discrimination threshold is changed.^(24,25)^ The AUROC value of 0.89 defined by the blue line of the ROC curve of figure 3 for the GBDT was slightly higher than that of AutoTriage, which yielded an AUROC of 0.88 (95% confidence interval 0.86 to 0.88)^(1)^ (Table 2).

ROC: Receiver Operating Characteristic.

**Table 2:** Comparison of the gradient boosted decision trees model’s performance with the AutoTriage system(1) and logistic regression model for the prediction of 12-hour mortality in the medical intensive care unit. The values for AutoTriage were obtained from Calvert et al.(1)

	Gradient-boosted decision trees^*^	AutoTriage^(1)^	Logistic regression^*^
Threshold	1.1222×10^−8^	-2	0.161
AUROC for mortality in the medical ICU	0.89	0.88	0.806
PPV	0.467	0.44	0.411
NPV	0.953	0.95	0.93
Sensitivity	0.801	0.80	0.701
Specificity	0.816	0.81	0.798
Diagnostic OR	17.823	16.26	9.254
Accuracy	0.814	0.80	0.782
Cohen's kappa	0.48		0.389
F-measure	0.59		0.518
MCC	0.509		0.412
MK	0.42		0.341

The threshold to calculate the accuracy statistics defines the cutoff value to consider the instance to be classified as positive (ICU death).

*Results presented are based on test set (n=1,979).

AUROC: area under the Receiver Operating Characteristic curve; ICU: intensive care unit; PPV: positive predictive value; NPV: negative predictive value; OR: odds ratio; MCC: Matthews correlation coefficient; MK: markedness.

For the GBDT, the PPV was 0.467, compared to 0.44 and 0.411 for AutoTriage and logistic regression, respectively. The NPV was 0.953 for the GBDT, *versus* 0.95 and 0.93 for AutoTriage and logistic regression, respectively. For the GBDT, the sensitivity was 0.801, compared to 0.80 and 0.701 for AutoTriage and logistic regression, respectively. The specificity was 0.816 for the GBDT, *versus* 0.81 and 0.798 for AutoTriage and logistic regression, respectively. The overall accuracy was 0.814 for the GBDT, compared to 0.80 and 0.782 for AutoTriage and logistic regression, respectively. The diagnostic OR was 17.823 for the GBDT, compared to 16.26 and 9.254 for AutoTriage and logistic regression, respectively (Tables 2 and 3). Gradient-boosted decision trees showed the greatest improvements in diagnostic OR and PPV (Table 2).

**Table 3:** Comparison of the gradient-boosted decision trees model’s performance with the logistic regression model for the prediction of 12-hour mortality in the medical intensive care unit, showing the accuracy statistics for the primary outcome (intensive care unit death) and the reference category

	Gradient-boosted decision trees		Logistic regression	
	
	Discharged	ICU death	Discharged	ICU death
PPV	0.953	0.467	0.93	0.411
NPV	0.467	0.953	0.411	0.93
Sensitivity	0.816	0.801	0.798	0.701
Specificity	0.801	0.816	0.701	0.798
Diagnostic OR	17.823	17.823	9.254	9.254
Accuracy	0.814	0.814	0.782	0.782
Cohen's kappa	0.48	0.48	0.389	0.389
F-measure	0.879	0.59	0.859	0.518
MCC	0.509	0.509	0.412	0.412
MK	0.42	0.42	0.341	0.341

PPV: positive predictive value; NPV: negative predictive value; OR: odds ratio; MCC: Matthews correlation coefficient; MK: markedness; ICU: intensive care unit.

The CK of GBDT was high, 0.48 (Tables 2 and 3). Cohen’s kappa values of 1 suggest a perfect agreement between the actual category and classifier models’ classification.^(24)^ It accounts for the chance of random classification of patients. The F-measure is defined as the weighted harmonic mean of the precision and recall of the test, with possible values ranging from 0 to 1. It was 0.59 and 0.518 for the GBDT and logistic regression, respectively (Tables 2 and 3).

The MCC is generally regarded as a balanced measure, and is a correlation coefficient value between -1 and +1, with +1 representing a perfect prediction.^(25)^ It was 0.509 and 0.412 for the GBDT and logistic regression, respectively (Tables 2 and 3). Markedness is a measure of reliability of PPV and NPV by a system, with its values ranging from -1 to +1. It also had the high value of 0.42 for the GBDT (Tables 2 and 3).

Simpler statistical models, such as the logistic regression, provide easy-to-understand models, while AI models demonstrate usually higher performance with reduced interpretability. If decision-making is to occur through the implantation of these AI algorithms, then it is necessary for physicians to understand the logic involved. Algorithms that explain patient-specific predictions have emerged that might increase the understanding of AI prediction models. The algorithm of Shapley additive explanations was applied to the GBDT model developed. It assigns to each feature a Shapley value that quantifies how much this particular feature changed the output, contributing to the deviation from the mean prediction of mortality.^(22)^

**Figure 4:**
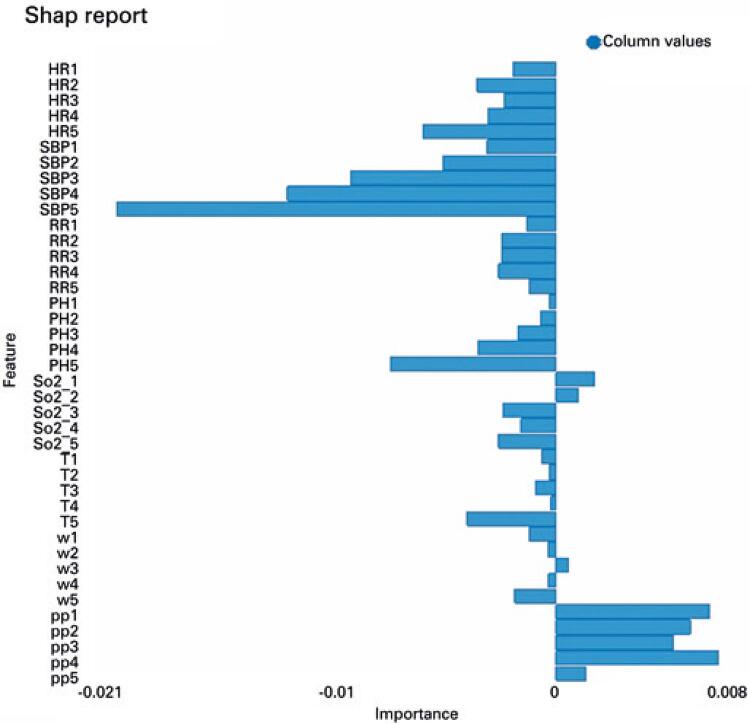
Algorithm of Shapley additive explanations for one individual patient. It represents the values for a correctly classified survivor by the gradient-boosted decision trees model. Shapley values are represented on the x-axis, showing how much each feature contributed to the probability of intensive care unit mortality for that patient. Features in the bars towards the right of zero favored mortality, whereas those towards the left favored survival

HR: heart rate; SBP: systolic blood pressure; RR: respiratory rate; PH: pH; So2: blood oxygen saturation; T: temperature; w: white blood cell count; pp: pulse pressure.

The ICU patient record whose prediction of ICU mortality was chosen to be explained corresponded to a patient that survived to ICU discharge. This patient was correctly predicted by the GBDT, which assigned a probability of discharge of 0.9999. The Shapley values are depicted in figure 4 for each feature related to the probability of mortality for that patient. As observed in figure 4, the pulse pressure of this patient contributed positively towards the probability of mortality, having the greatest contribution towards mortality in the context of the other features. Most features pull towards survival with negative Shapley values. For example, the systolic blood pressure and heart rate of this patient contributed more towards survival in comparison to the other features.

## DISCUSSION

The GBDT model developed was able to identify individual patients at risk for all-factor 12-hour mortality at the medical ICU, using data extracted from 5 consecutive hours of a patient’s medical ICU stay. The results were compared with the AutoTriage system^(1)^ and a logistic regression model built on the same platform.

When comparing the results from the AUROC, it was observed that it was higher for the GBDT. Overall, the nature of the ROC plot and the high AUROC values of the GBDT and AutoTriage^(1)^ indicate that the discriminatory power was excellent for both.

The slightly higher PPV for GBDT of 0.467 means fewer false positive results. This is important for a predictor at the ICU, indicating a lower rate of false alarms, which can decrease alarm fatigue and increase the confidence in a mortality prediction. This PPV is influenced by the low prevalence of ICU mortality in the study cohort. The slightly higher PPV for GBDT was achieved despite the studied cohort having an even slightly lower prevalence of mortality (15.5059% *versus* 16.26% for the AutoTriage cohort).^(1)^ The NPV was high for GBDT and AutoTriage. Positive predictive value and NPV are dependent on mortality prevalence.

The GBDT showed a higher accuracy of 0.814 in comparison to the other models. Though accuracy provides an overall assessment of the performance of the classifiers, one limitation to the use of accuracy is “accuracy paradox”.^(24)^ Additionally, accuracy is also dependent on mortality prevalence. Therefore, less biased metrics were utilized as a more objective analyzer. The CK, F-measure and MCC values of the GBDT (Table 2), which were also high, support the good predictive power of the GBDT.

Most importantly, it must be noted the high values obtained of diagnostic OR and MK for the GBDT. The higher diagnostic OR value of 17.823 was obtained for GBDT. A high MK value of 0.42 was obtained for GBDT. These two measures, diagnostic OR and MK, have been recommended as the best options to evaluate on unbalanced datasets, as in this cohort, being among the least sensitive measures to dataset composition.^(25)^

The GBDT developed in this work could be applied continuously for an individual patient. New predictions could be calculated during the ICU stay. This is supported using frequently measured routine patient clinical variables.

Although it is understandable that a complex model such as the GBDT can show higher performance than the logistic regression, improved performance of AI has the drawback of difficulty in interpretability. Understanding the reasoning behind AI predictions is very important for physicians. The explainer algorithm applied in this work provides understanding of how the GBDT arrived at the predictions. Figure 4 displays the contribution of the feature variables to the patient-specific mortality prediction in a way that is visually explainable.

Limitations of this work include the consideration that the dataset was collected from a single organization. For general applicability of the method, the GBDT should be tested on data from a different institution. However, demographically diverse patient populations could result in performance variability. Data from different regions may be of a diverse nature, with differences in the incidence of ICU mortality. Training the model on data from each organization could improve the performance in these cases.

The computational costs of this AI model are mainly related to background processing (Table 4).

**Table 4:** The computational costs of the artificial intelligence model infrastructure developed on an open-source platform

Steps	Computational costs	
Training on a new population	Dependent on the database system used and the speed of queries. In this model, a PostgreSQL database management system was used	Optimization strategies are targeted at the size of the tables in bytes, indexing, CPU cores, using a cloud-based instance of the database, etc.
Testing for the generation of the hourly predictions	Generated instantly as long as the data are collected from an intensive care information system which is interfaced with the patient monitors, blood gas analysis devices and laboratories	

CPU: central processing unit.

It should be recognized that the algorithm results exclusively apply to patients that are still at the ICU after 17 hours. Patients that are discharged or die before reaching an ICU stay of 17 hours are not investigated. The algorithm may perform differently in these patients, but the model is thought not to be used in those patients with length of stay shorter than 17 hours.

The patients who had more than one ICU stay during a hospitalization were also included in the study cohort. This could be a potential source of bias. Nonetheless, addressing this by selecting the first ICU stay, for instance, makes it much more difficult to compare to the AutoTriage algorithm, which included ICU re-admissions.

## CONCLUSION

From the results of the metrics used for evaluation and the parameter values provided by the optimization loop, it could be concluded that the gradient-boosted decision trees model showed higher performance than the logistic regression model, compared in terms of predicting 12-hour mortality at the medical intensive care unit. The excellent performance of the gradient-boosted trees was achieved despite the cohort being an unbalanced dataset, and highlights the usability and flexibility of artificial intelligence models with few patient features for mortality prediction at the medical intensive care unit, to assist physicians to monitor patients with critical conditions.
