# Supplementary material for: Artificial intelligence forecasting mortality at an intensive care unit and comparison to a logistic regression system
Source: Einstein (Sao Paulo). 2021 Sep 29;19:eAO6283. doi: 10.31744/einstein_journal/2021AO6283 (PMC8483638; doi:10.31744/einstein_journal/2021AO6283)
Supplement: Translation

# Translation: Inteligência artificial que prevê a mortalidade em uma unidade de terapia intensiva e comparação com um sistema de regressão logística

**Authors:** Beatriz Nistal-Nuño

**Affiliations:** 1 Spain Departamento de Anestesiologia, Complexo Hospitalario Universitario de Pontevedra, Pontevedra, PO, Spain.

**Keywords:** Inteligência artificial, Árvores de decisão impulsionadas por gradiente, Unidades de terapia intensiva, Banco de dados MIMIC-III, Mortalidade, Discriminação, Regressão logística

## Abstract

**Objetivo:**

Explorar uma abordagem de inteligência artificial baseada em árvores de decisão impulsionadas por gradiente para previsão de mortalidade por todas as causas em unidade de terapia intensiva, comparando seu desempenho com um sistema de regressão logística recente na literatura e um modelo de regressão logística construído na mesma plataforma.

**Métodos:**

Foram desenvolvidos um modelo de árvores impulsionadas por gradiente e um modelo de regressão logística, treinados e testados com o banco de dados *Medical Information Mart for Intensive Care*. As medidas fisiológicas de pacientes adultos com resolução de 1 hora, coletadas durante 5 horas na unidade de terapia intensiva, consistiram em oito parâmetros clínicos de rotina. Estudou-se como os modelos aprendem a categorizar os pacientes para prever a mortalidade ou a sobrevida, em unidades de terapia intensiva, em 12 horas. O desempenho foi avaliado por meio de estatísticas de acurácia e pela área sob a curva Característica de Operação do Receptor.

**Resultados:**

As árvores impulsionadas por gradiente produziram área sob a curva Característica de Operação do Receptor de 0,89, em comparação com 0,806 para a regressão logística. A acurácia foi de 0,814 para as árvores impulsionadas por gradiente, em comparação com 0,782 para a regressão logística. A razão de chances de diagnóstico foi de 17,823 para as árvores impulsionadas por gradiente, em comparação a 9,254 para a regressão logística. O kappa de Cohen, a medida F, o coeficiente de correlação de Matthews e a marcação foram maiores para as árvores impulsionadas por gradiente.

**Conclusão:**

O poder discriminatório das árvores impulsionadas por gradiente foi excelente. As árvores impulsionadas por gradiente superaram a regressão logística em relação à previsão de mortalidade em unidade de terapia intensiva. A alta razão de chances de diagnóstico e os valores de marcação para as árvores impulsionadas por gradiente são importantes no contexto do conjunto de dados não balanceados estudado.

## INTRODUÇÃO

A previsão precisa e oportuna da mortalidade do paciente antes de sua rápida deterioração pode ser fundamental, especialmente em uma unidade de terapia intensiva (UTI).^(1)^ A deterioração das variáveis fisiológicas e bioquímicas frequentemente precede a deterioração clínica dos pacientes nessa unidade.^(2)^ A previsão de mortalidade na UTI permite que sejam realizadas intervenções precoces para remediar situações clínicas iminentes, que poderiam conduzir a um evento crítico e ao óbito.^(1)^

A fim de antecipar a deterioração do paciente na UTI, foram desenvolvidos vários escores de gravidade da doença. O *Acute Physiology and Chronic Health Evaluation* (APACHE) II fornece previsões de mortalidade de pacientes com base em dados coletados na UTI,^(3)^ tendo sido aprimorado para o APACHE III, em 1991.^(4)^ Uma nova versão foi publicada em 2006, o APACHE IV, que acrescentou novas variáveis e aplicou um método estatístico diferente.^(5)^O *Simplified Acute Physiology Score* (SAPS) II foi criado para avaliar a gravidade da doença em pacientes com 15 anos de idade ou mais internados em UTI.^(6)^ O posterior, SAPS III, é um suplemento do SAPS II.^(7)^

O escore *Sequential Organ Failure Assessment* (SOFA) é utilizado para analisar as condições de um paciente durante sua permanência na UTI e o grau de função de seus órgãos.^(8)^ Esse escore é baseado em seis escores diferentes, sendo um para cada sistema: nervoso central, cardiovascular, respiratório, renal, hepático e de coagulação. No *Logistic Organ Dysfunction System* (LODS), as variáveis fisiológicas avaliam a disfunção também em seis sistemas de órgãos.^(9)^ O *Oxford Acute Severity of Illness Score* foi desenvolvido por Johnson et al.^(10)^ O sistema *Mortality Prediction Model* (MPM)-II calcula a probabilidade de mortalidade hospitalar para pacientes em UTI.^(11)^

A maioria desses sistemas de predição são de pontuação linear, com base em uma combinação linear ponderada de características do paciente.^(1)^ Essas ferramentas de predição pressupõem que as características do paciente não estejam relacionadas entre si e, consequentemente, não podem captar a complexa fisiologia inter-relacionada dos pacientes.^(1)^ Os modelos de predição para UTIs, como APACHE, MPM, LODS e SAPS II e III, são baseados em regressão logística multivariável.^(12)^ Foram desenvolvidos métodos estatísticos aprimorados a esse respeito, como o sistema recente de Calvert et al.,^(1)^ que avalia as correlações entre variáveis preditoras clínicas agrupadas com mortalidade por todas as causas em 12 horas na UTI, além de realizar a análise da tendência temporal das medidas dos pacientes por meio de regressão logística.

Uma das razões para o baixo poder preditivo de muitos dos sistemas de pontuação estabelecidos aqui mencionados reside na não normalidade e na não linearidade das variáveis envolvidas na modelagem, bem como nas relações não lineares entre as variáveis fisiológicas e o *log odds* do desfecho, quando a regressão logística é usada.

A inteligência artificial (IA) tem se mostrado útil nesse contexto, sendo um método promissor para avaliar a mortalidade em UTI.^(12-14)^ Johnson et al., desenvolveram um método de predição de mortalidade em UTI usando um novo algoritmo de aprendizagem por conjunto bayesiano. O método de predição proposto teve um desempenho favorável, com o potencial de ser utilizado com sucesso para predições de pacientes individuais.^(15)^

Johnson et al., compararam a IA, na forma de árvores de decisão impulsionadas por gradiente (GBDT - *gradient-boosted decision trees*), com vários tipos de regressão logística e modelos da literatura para previsão em tempo real da mortalidade de pacientes em UTI. A GBDT apresentou a maior área sob a curva Característica de Operação do Receptor (ASC COR).^(16)^ Darabi et al., aplicaram a GBDT e as redes neurais profundas para estimar o risco de mortalidade de pacientes em UTI.^(17)^

Kim et al., avaliaram se o desempenho de várias técnicas de IA, como rede neural artificial, máquina de vetores de suporte e árvores de decisão (DTs - *decision trees*), superava a regressão logística convencional para previsão de mortalidade em UTI e descobriram que o algoritmo DT superava ligeiramente as outras técnicas.^(18)^

Um método de IA aplicado com sucesso nesse contexto é a GBDT. Esta pesquisa se baseia em trabalho anterior de Calvert et al.,^(1)^ mas usa a GBDT para comparar os resultados.

## OBJETIVO

Explorar uma abordagem de inteligência artificial que utiliza árvores impulsionadas por gradiente para previsão de mortalidade por todas as causas, em unidades de terapia intensiva, usando o banco de dados *Medical Information Mart for Intensive Care III*, comparando o desempenho das árvores impulsionadas por gradiente com um modelo de regressão logística construído na mesma plataforma e o sistema *AutoTriage* para previsão de mortalidade em 12 horas, nessas unidades. Até o momento, essa comparação ainda não havia sido estudada.

## MÉTODOS

### População de pacientes e extração de dados

Foi utilizado o grande banco de dados de terapia intensiva *Medical Information Mart for Intensive Care* (MIMIC) III, versão v1.4., que contém dados clínicos individuais abrangentes não identificados de pacientes internados nas UTIs de um grande hospital terciário, o *Beth Israel Deaconess Medical Center*, em Boston, Estados Unidos.^(19)^ O MIMIC III contém dados de pacientes adultos admitidos em UTIs entre 2001 e 2012.^(19,20)^

Foi utilizado, neste estudo, um conjunto final de dados de 9.893 prontuários de pacientes internados em UTI do banco de dados MIMIC-III, que foram selecionados de acordo com as etapas de extração de dados descritas na figura 1. O processo de exclusão de pacientes foi realizado do modo mais semelhante possível ao realizado por Calvert et al.,^(1)^ para comparar os resultados com o sistema *AutoTriage*. O subconjunto selecionado continha os registros de permanência em UTI de pacientes adultos com idade de 18 anos ou mais, admitidos em UTIs médica, com pelo menos uma observação de cada medição dos parâmetros específicos usados nas análises e tempos de internação e sobrevida de 17 horas a 500 horas, após a admissão.

**Figura 1:** Etapas de extração de dados de pacientes do banco de dados *Medical Information Mart for Intensive Care III*

MIMIC III: *Medical Information Mart for Intensive Care III*; UTI: unidade de terapia intensiva.

O número de registros de internação em UTI extraídos em cada etapa é igual ao do estudo,^(1)^ exceto nas duas últimas etapas. Isso se deve ao fato de que, no estudo atual, somente foram coletadas medidas de temperatura em graus Celsius, e este estudo utiliza uma versão posterior do banco de dados MIMIC-III. Assim, não foi possível extrair exatamente o mesmo número de registros de internação em UTI para a etapa 4, o que afetou também a etapa 5. No entanto, a diferença no número final de registros de internação em UTI para análise é muito pequena, de apenas 210 internações em UTI do número final de 9.893 registros de internação em UTI coletados para este estudo. O código desenvolvido em linguagem PostgreSQL para seleção das internações em UTI está disponível em https://doi.org/10.7910/DVN/UMJVWA.^(21)^

Dos 9.893 registros finais de internação em UTI selecionados para análise, 1.534 resultaram em óbito durante a internação na UTI, e 8.359 resultaram em alta da UTI com sobrevida. Isso equivale à prevalência de 15,5059% de mortalidade na UTI.

### Fatores associados à mortalidade

As medições fisiológicas de resolução de 1 hora coletadas durante 5 horas consecutivas nos 9.893 registros de internação em UTI foram frequência cardíaca, pH, pressão de pulso, frequência respiratória, saturação de oxigênio no sangue, pressão arterial sistólica, temperatura e contagem de leucócitos. Essas oito variáveis foram escolhidas com base no estudo de Calvert et al.,^(1)^ a fim de tornar os resultados atuais comparáveis e por serem parâmetros clínicos de rotina frequentemente medidos em UTI.

O pré-processamento de dados foi realizado com base no conhecimento do domínio para remover registros errôneos, como valores fisiologicamente inválidos e erros de unidade. Para um único valor por hora ausente, uma substituição foi calculada como sendo o valor disponível imediatamente anterior na janela de 5 horas. Para um valor ausente na primeira medição por hora, uma substituição foi calculada como sendo o valor disponível imediatamente posterior na janela de 5 horas. Para as internações em UTI em que nenhum dado foi coletado para um determinado parâmetro durante a janela de 5 horas, os valores aplicados foram os da faixa normal para aquele parâmetro.

Esses dados foram importados para o *Konstanz Information Miner* (KNIME), versão 4.2.0 (KNIME AG, Zurique, Suíça),^(22)^ no qual foram implementados os modelos GBDT e regressão logística para executar as simulações. Estudou-se como tais modelos aprendiam a representar e categorizar esses pacientes, com base em suas características selecionadas nas categorias de óbito na UTI ou alta da UTI, em um momento ocorrido 12 horas antes do óbito ou alta do paciente.

O conjunto de dados de entrada foi dividido aleatoriamente em duas partes: 80% para dados de treinamento e 20% para dados de teste. Isso ocorreu depois que o nó do KNIME normalizou os valores de todas as variáveis de entrada numéricas por normalização do escore-Z (gaussiana) (Figura 2).

**Figura 2:** Fluxo de trabalho do *Konstanz Information Miner*. Captura de tela do fluxo de trabalho do *Konstanz Information Miner* usado para construir as árvores de decisão impulsionadas por gradiente e o modelo de regressão logística

ASC COR: área sob a curva Característica de Operação do Receptor.

### Simulações de aprendizagem de inteligência artificial

O método de aprendizado de conjunto de IA usado envolve uma combinação de vários modelos de IA de algoritmos de aprendizado supervisionados para obter um modelo geral mais preciso. A técnica de conjunto usada é o *boosting*.^(22)^ As GBDT são um modelo de conjunto que combina múltiplas DTs sequenciais simples em um modelo mais robusto, usando uma forma especial de *boosting*. A cada iteração, um DT simples é ajustado para prever os resíduos do modelo atual, seguindo o gradiente da função de perda, e é adicionado ao conjunto para melhorar os resultados do estado do modelo anterior, levando a um melhor desempenho após cada iteração.^(22)^ A implementação segue o algoritmo descrito em Friedman.^(23)^

Foi implementado no KNIME pelo nó aprendiz GBDT e pelo nó preditor GBDT (Figura 2). A GBDT tem os parâmetros mencionados na tabela 1, que são otimizados por meio de otimização de parâmetros.^(22)^

**Tabela 1:** Melhores parâmetros encontrados durante os *loops* de otimização de parâmetros para o modelo de árvores de decisão impulsionadas por gradiente e o modelo de regressão logística

Parâmetros	Árvores de decisão impulsionadas por gradiente	Regressão logística
Profundidade da árvore	7	
Número de modelos (DTs) para aprender	1.175	
Taxa de aprendizagem	0,1	
Fração de registros de UTI para cada DT individual	0,5	
Amostragem de atributos (fração linear) de características do paciente por nó de árvore	0,1	
*Solver*		Mínimos quadrados iterativamente reponderados
Número máximo de épocas		2.140
*Epsilon*		0,01
Número máximo de iterações^*^	271	791
Número de rodadas para interrupção precoce^*^	108	188

^*^ Os dois últimos parâmetros são para o algoritmo de otimização de parâmetros e não para o projeto dos modelos.

DT: árvore de decisão; UTI: unidade de terapia intensiva.

### Simulações de aprendizagem de regressão logística

A regressão logística é um algoritmo estatístico que modela a relação entre os recursos de entrada e as classes de saída categóricas, maximizando uma função de verossimilhança.^(22)^ Neste estudo, ela foi construída para o problema binário na mesma plataforma, de modo a ser comparada com o modelo de IA desenvolvido, além da comparação feita com o sistema *AutoTriage.*^(1)^

Foi implementada no KNIME pelo nó aprendiz regressão logística e pelo nó preditor regressão logística (Figura 2). A regressão logística tem os parâmetros mencionados na tabela 1, que são otimizados por meio da otimização dos parâmetros.^(22)^

Foi utilizada a técnica de otimização de parâmetros com um *loop* de otimização de parâmetros para encontrar os parâmetros ótimos para os modelos GBDT e regressão logística. Isso foi implementado no KNIME com o nó inicial do *loop* de otimização de parâmetros e o nó final do *loop* de otimização de parâmetros (Figura 2).

Os parâmetros mencionados na tabela 1, controlados por meio de variáveis de fluxo, foram escolhidos por um algoritmo para maximizar a ASC COR para o desfecho de predição de mortalidade na UTI.^(22)^ Os melhores valores dos parâmetros encontrados durante os *loops* após várias simulações de otimização estão mostrados na tabela 1. Os parâmetros restantes foram definidos com seus valores padrão.

### Medidas de desempenho

As métricas para avaliar os modelos GBDT e regressão logística e compará-los com o *AutoTriage* foram várias estatísticas de acurácia e a curva COR com a ASC COR. Essas medidas foram obtidas após a predição da classe de desfecho de 12 horas do conjunto de teste, depois do treinamento dos modelos com o conjunto de treinamento. As estatísticas de acurácia avaliadas foram valor preditivo positivo (VPP), valor preditivo negativo (VPN), sensibilidade, especificidade, razão de chances (RC) de diagnóstico, acurácia geral, kappa de Cohen (CK), medida F, coeficiente de correlação de Matthews (MCC) e marcação (MK).

## RESULTADOS

A figura 3 mostra as curvas COR dos classificadores GBDT e regressão logística, que correspondem aos valores da ASC COR de 0,89 e 0,806, respectivamente (Tabela 2). A curva COR é uma representação gráfica que mostra o desempenho de um classificador binário conforme seu limite de discriminação é alterado.^(24,25)^ O valor da ASC COR de 0,89 definido pela linha azul da curva COR da figura 3 para a GBDT foi ligeiramente superior ao *AutoTriage*, o que resultou em uma ASC COR de 0,88 (intervalo de confiança de 95% de 0,86 a 0,88)^(1)^ (Tabela 2).

**Figura 3:** Curva Característica de Operação do Receptor para previsão de mortalidade em 12 horas em unidades de terapia intensiva médica, para as árvores de decisão impulsionadas por gradiente e modelo de regressão logística desenvolvidos. O valor que representa se o paciente foi a óbito na unidade de terapia intensiva ou recebeu alta após o intervalo de 12 horas foi representado pela variável-alvo de duas classes *icustay_expire_flag*

COR: Característica de Operação do Receptor.

**Tabela 2:** Comparação do desempenho do modelo de árvores de decisão impulsionadas por gradiente com o do sistema *AutoTriage*(1) e o modelo de regressão logística para a previsão de mortalidade de 12 horas em unidades de terapia intensiva médica. Os valores para *AutoTriage* foram obtidos de Calvert et al.(1)

	Árvores de decisão impulsionadas por gradiente^*^	*AutoTriage* ^(1)^	Regressão logística^*^
Limite	1,1222×10^−8^	-2	0,161
ASC COR para mortalidade em UTI médica	0,89	0,88	0,806
VPP	0,467	0,44	0,411
VPN	0,953	0,95	0,93
Sensibilidade	0,801	0,80	0,701
Especificidade	0,816	0,81	0,798
RC diagnóstica	17,823	16,26	9,254
Acurácia	0,814	0,80	0,782
Kappa de Cohen	0,48		0,389
Medida F	0,59		0,518
MCC	0,509		0,412
MK	0,42		0,341

O limite para calcular as estatísticas de acurácia define o valor de corte para considerar a instância a ser classificada como positiva (óbito na UTI).

^*^Os resultados apresentados são baseados no conjunto de teste (n=1.979).

ASC COR: área sob a curva Característica de Operação do Receptor; UTI: unidade de terapia intensiva; VPP: valor preditivo positivo; VPN: valor preditivo negativo; RC: razão de chances; MCC: coeficiente de correlação de Matthews; MK: marcação.

Para a GBDT, o VPP foi de 0,467, em comparação com 0,44 e 0,411 para *AutoTriage* e regressão logística, respectivamente. O VPN foi de 0,953 para a GBDT, contra 0,95 e 0,93 para *AutoTriage* e regressão logística, respectivamente. Para a GBDT, a sensibilidade foi de 0,801, comparada a 0,80 e 0,701 para *AutoTriage* e regressão logística, respectivamente. A especificidade foi de 0,816 para a GBDT, contra 0,81 e 0,798 para *AutoTriage* e regressão logística, respectivamente. A acurácia geral foi de 0,814 para a GBDT, em comparação com 0,80 e 0,782 para *AutoTriage* e regressão logística, respectivamente. A RC diagnóstica foi de 17,823 para a GBDT, em comparação com 16,26 e 9,254 para *AutoTriage* e regressão logística, respectivamente (Tabelas 2 e 3). As GBDTs mostraram as maiores melhorias em RC diagnóstica e VPP (Tabela 2).

**Tabela 3:** Comparação do desempenho do modelo de árvores de decisão impulsionadas por gradiente com o modelo de regressão logística para a previsão de mortalidade em 12 horas em unidades de terapia intensiva médica, mostrando as estatísticas de acurácia para o desfecho primário (óbito na unidade de terapia intensiva) e a categoria de referência

	Árvores de decisão impulsionadas por gradiente		Regressão logística	
	
	Alta	Óbito na UTI	Alta	Óbito na UTI
VPP	0,953	0,467	0,93	0,411
VPN	0,467	0,953	0,411	0,93
Sensibilidade	0,816	0,801	0,798	0,701
Especificidade	0,801	0,816	0,701	0,798
RC diagnóstica	17,823	17,823	9,254	9,254
Acurácia	0,814	0,814	0,782	0,782
Kappa de Cohen	0,48	0,48	0,389	0,389
Medida F	0,879	0,59	0,859	0,518
MCC	0,509	0,509	0,412	0,412
MK	0,42	0,42	0,341	0,341

UTI: unidade de terapia intensiva; VPP: valor preditivo positivo; VPN: valor preditivo negativo; RC: razão de chances; MCC: coeficiente de correlação de Matthews; MK: marcação.

O CK da GBDT foi alto – 0,48 (Tabelas 2 e 3). O valor de CK de 1 sugere uma concordância perfeita entre a categoria real e a classificação dos modelos classificadores.^(24)^ É responsável pela chance de classificação aleatória dos pacientes. A medida F é definida como a média harmônica ponderada da precisão e sensibilidade, com valores possíveis variando de 0 a 1. Foi de 0,59 e 0,518 para a GBDT e regressão logística, respectivamente (Tabelas 2 e 3).

O MCC é geralmente considerado uma medida balanceada e é um valor de coeficiente de correlação entre -1 e +1, com +1 representando previsão perfeita.^(25)^ Foi 0,509 e 0,412 para a GBDT e regressão logística, respectivamente (Tabelas 2 e 3). A MK é uma medida de confiabilidade das previsões positivas e negativas de um sistema, com seus valores variando de -1 a +1. Teve também o valor alto de 0,42 para a GBDT (Tabelas 2 e 3).

Os modelos estatísticos mais simples, como a regressão logística, fornecem modelos fáceis de entender, ao passo que os modelos de IA demonstram desempenho geralmente superior com interpretabilidade reduzida. Para que a tomada de decisão ocorra por meio da implantação desses algoritmos de IA, é necessário que os médicos entendam a lógica envolvida. Surgiram algoritmos que explicam as previsões específicas em relação aos pacientes, que podem aumentar a compreensão dos modelos de previsão de IA. O algoritmo de explicações aditivas de Shapley foi aplicado ao modelo GBDT desenvolvido. Ele atribui a cada característica um valor de Shapley, que quantifica o quanto essa particularidade alterou o resultado, contribuindo para o desvio da previsão média de mortalidade.^(22)^

O prontuário do paciente cuja previsão de mortalidade na UTI foi escolhida para ser explicada correspondeu a um paciente que sobreviveu e teve alta da UTI. Esse paciente foi corretamente previsto pela GBDT, que atribuiu probabilidade de alta de 0,9999. Os valores de Shapley são representados na figura 4 para cada característica referente à probabilidade de mortalidade para aquele paciente. Como se pode observar na figura 4, a pressão de pulso desse paciente contribuiu positivamente para a probabilidade de mortalidade, tendo a maior contribuição para a mortalidade no contexto das demais características. A maioria das características tendeu para a sobrevida com valores Shapley negativos. Por exemplo, a pressão arterial sistólica e a frequência cardíaca desse paciente contribuíram mais para a sobrevida em comparação com as outras características.

**Figura 4:** Algoritmo de explicações aditivas de Shapley para um paciente individual. Representa os valores para um sobrevivente classificado corretamente pelo modelo de árvores de decisão impulsionadas por gradiente. Os valores Shapley são representados no eixo x, mostrando o quanto cada característica contribuiu para a probabilidade de mortalidade na unidade de terapia intensiva para aquele paciente. As características das barras à direita do zero favorecem a mortalidade, enquanto aquelas à esquerda do zero favorecem a sobrevida

HR: *heart rate*; SBP: *systolic blood pressure*; RR: *respiratory rate*; PH: pH; So2: *blood oxygen saturation*; T: *temperature*; w: *white blood cell count*; pp: *pulse pressure*.

## DISCUSSÃO

O modelo GBDT desenvolvido foi capaz de identificar pacientes individuais em risco de mortalidade de 12 horas por todos os fatores em UTI médica, usando dados extraídos de 5 horas consecutivas de permanência do paciente na unidade. Os resultados foram comparados com os do sistema *AutoTriage*^(1)^ e um modelo regressão logística construído na mesma plataforma.

Quando comparados os resultados da ASC COR, observou-se que eles foram superiores para a GBDT. No geral, a natureza do gráfico COR e os altos valores da ASC COR da GBDT e *AutoTriage*^(1)^indicam que o poder discriminatório foi excelente para ambos.

O VPP ligeiramente superior para GBDT de 0,467 significa menos falsos-positivos. Isso é importante para um preditor na UTI, indicando taxa menor de alarmes falsos, o que pode diminuir a fadiga do alarme e aumentar a confiança em uma previsão de mortalidade. Esse VPP é influenciado pela baixa prevalência de mortalidade em UTI na coorte do estudo. O VPP ligeiramente mais alto para GBDT foi alcançado apesar da coorte estudada ter prevalência de mortalidade ainda ligeiramente mais baixa (15,5059% *versus* 16,26% para a coorte do *AutoTriage).*^(1)^ O VPN foi alto para GBDT e *AutoTriage*. O VPP e o VPN são dependentes da prevalência de mortalidade.

A GBDT apresentou maior acurácia de 0,814 em comparação com os demais modelos. Embora a acurácia forneça uma avaliação geral do desempenho dos classificadores, uma limitação do uso da acurácia é o “paradoxo da acurácia”.^(24)^ Além disso, a acurácia também depende da prevalência de mortalidade. Portanto, métricas menos tendenciosas foram utilizadas como um analisador mais objetivo. Os valores de CK, medida F e MCC da GBDT (Tabela 2), também elevados, apoiam o bom poder preditivo da GBDT.

Mais importante ainda, devem ser destacados os elevados valores obtidos de RC diagnóstica e MK para GBDT. O maior valor RC diagnóstica de 17,823 foi obtido para GBDT. Um alto valor de MK de 0,42 foi obtido para GBDT. Essas duas medidas RC diagnóstica e MK têm sido recomendadas como as melhores opções para avaliar conjuntos de dados não balanceados, como nesta coorte, estando entre as medidas menos sensíveis para a composição do conjunto de dados.^(25)^

A GBDT desenvolvida neste estudo pode ser aplicada continuamente para um paciente individual. Novas previsões poderiam ser calculadas durante a permanência na UTI. Isso é apoiado pelo uso de variáveis clínicas de rotina do paciente medidas com frequência.

Embora seja compreensível que um modelo complexo, como a GBDT, possa apresentar desempenho superior ao da regressão logística, o desempenho aprimorado da IA tem a desvantagem da dificuldade de interpretabilidade. É muito importante para os médicos compreender o raciocínio por trás das previsões de IA. O algoritmo explicador aplicado neste estudo proporciona entendimento de como a GBDT chegou às previsões. Está mostrada na figura 4 a contribuição das variáveis de características para a previsão de mortalidade específica do paciente de uma forma que seja visualmente explicável.

As limitações deste estudo incluem a consideração de que o conjunto de dados foi coletado de uma única instituição. Para uma aplicabilidade geral do método, a GBDT deve ser testada em dados de uma instituição diferente. No entanto, populações de pacientes demograficamente diversas podem resultar em variabilidade de desempenho. Os dados de muitas regiões podem ser de natureza diversa, com diferenças na incidência de mortalidade na UTI. O desempenho nesses casos pode ser melhorado treinando o modelo com os dados de cada instituição.

Os custos computacionais deste modelo de IA estão principalmente relacionados ao processamento de fundo (Tabela 4).

**Tabela 4:** Custos computacionais de infraestrutura do modelo de inteligência artificial desenvolvido em plataforma *open source*

Etapas	Custos computacionais	
Treinamento em uma nova população	Depende do sistema de banco de dados utilizado e da velocidade das consultas de código. Nesse modelo, foi utilizado um sistema de gerenciamento de banco de dados PostgreSQL	As estratégias de otimização devem ser direcionadas ao tamanho das tabelas em *bytes*, indexação, núcleos de CPU, usando uma instância de banco de dados com base em nuvem, etc.
Teste para geração das previsões a cada hora	Gerado instantaneamente, desde que os dados sejam coletados de um sistema de informação de unidades de terapia intensiva que faça interface com os monitores dos pacientes e com os dispositivos de análise de gases sanguíneos e laboratórios	

CPU: *central processing unit.*

Deve-se reconhecer que os resultados do algoritmo se aplicam exclusivamente aos pacientes que ainda estiverem na UTI após 17 horas. Os pacientes que recebem alta ou vão a óbito antes de completarem 17 horas de internação na UTI não são investigados. O algoritmo pode ter um desempenho diferente nesses pacientes, mas o modelo não deve ser usado em pacientes com tempo de internação inferior a 17 horas.

Os pacientes que tiveram mais de uma internação em UTI durante a hospitalização também foram incluídos na coorte do estudo. Isso pode ser uma possível fonte de viés. Porém, ao se abordar isso, por exemplo, selecionando a primeira internação na UTI, seria ainda mais dificultada a comparação com o algoritmo do *AutoTriage*, que incluía reinternações na UTI.

## CONCLUSÃO

A partir dos resultados das métricas utilizadas para avaliação e dos valores dos parâmetros fornecidos pelo *loop* de otimização, pode-se concluir que o modelo de árvores de decisão impulsionadas por gradiente apresentou desempenho superior, em comparação com o modelo de regressão logística na previsão de mortalidade de 12 horas em unidades de terapia intensiva médica. O excelente desempenho das árvores impulsionadas por gradiente foi alcançado, apesar de a coorte ser um conjunto de dados não balanceados, destacando a usabilidade e a flexibilidade dos modelos de inteligência artificial com poucas características dos pacientes para a previsão da mortalidade em unidades de terapia intensiva médica, de modo a ajudar os médicos a monitorar pacientes em condições críticas.
